# Genomic and Transcriptomic Evidence for Carbohydrate Consumption among Microorganisms in a Cold Seep Brine Pool

**DOI:** 10.3389/fmicb.2016.01825

**Published:** 2016-11-15

**Authors:** Weipeng Zhang, Wei Ding, Bo Yang, Renmao Tian, Shuo Gu, Haiwei Luo, Pei-Yuan Qian

**Affiliations:** ^1^Division of Life Science, Hong Kong University of Science and TechnologyHong Kong, Hong Kong; ^2^Simon F. S. Li Marine Science Laboratory, School of Life Sciences, Chinese University of Hong KongShatin, Hong Kong

**Keywords:** brine pool, biofilm, carbon metabolism, microbial genomics, transcriptomics

## Abstract

The detailed lifestyle of microorganisms in deep-sea brine environments remains largely unexplored. Using a carefully calibrated genome binning approach, we reconstructed partial to nearly-complete genomes of 51 microorganisms in biofilms from the Thuwal cold seep brine pool of the Red Sea. The recovered metagenome-assembled genomes (MAGs) belong to six different phyla: Actinobacteria, Proteobacteria, *Candidatus* Cloacimonetes, *Candidatus* Marinimicrobia, Bathyarchaeota, and Thaumarchaeota. By comparison with close relatives of these microorganisms, we identified a number of unique genes associated with organic carbon metabolism and energy generation. These genes included various glycoside hydrolases, nitrate and sulfate reductases, putative bacterial microcompartment biosynthetic clusters (BMC), and F_420_H_2_ dehydrogenases. Phylogenetic analysis suggested that the acquisition of these genes probably occurred through horizontal gene transfer (HGT). Metatranscriptomics illustrated that glycoside hydrolases are among the most highly expressed genes. Our results suggest that the microbial inhabitants are well adapted to this brine environment, and anaerobic carbohydrate consumption mediated by glycoside hydrolases and electron transport systems (ETSs) is a dominant process performed by microorganisms from various phyla within this ecosystem.

## Introduction

Microorganisms play important roles in the biogeochemical cycles of deep-sea environments, such as deep-sea hydrothermal vents and brine pools. Studies of microbial lifestyles in brine pools are less common than those of hydrothermal vents. The brine pools studied so far include the Shaban Deep (Ferrer et al., 2012), Discovery Deep (Wang et al., 2013), Atlantis II Deep (Ngugi et al., 2015), and Kebrit Deep (Guan et al., 2015) of the Red Sea, which contains more than 25 deep hypersaline anoxic pools (Antunes et al., 2011). Previous investigations of other deep-sea brine pools included those in the Mediterranean Sea (van der Wielen et al., 2005) and the Gulf of Mexico (Formolo and Lyons, 2013). Brine pools probably share a common formation process of tectonically induced brine formation, resulting in frequently observed high methane and sulfite fluxes (Faber et al., 1998). As a consequence, the prevalent processes in the microbial inhabitants are anaerobic oxidation of methane and sulfite and sulfate reduction (Antunes et al., 2011). Surveys of brine pools identified a few widespread groups including Proteobacteria, Actinobacteria, Cyanobacteria, and Deferribacteres, and showed a higher diversity of bacteria over the archaea (Ferrer et al., 2012; Guan et al., 2015; Ngugi et al., 2015). Despite the common origin and shared features of the investigated brine pools, regional variation may lead to distinct combinations of physicochemical parameters and sustains microorganisms with unique adaptive strategies.

As important components of marine ecosystems, biofilms play an important role in ocean carbon transformation (Bhaskar and Bhosle, 2005; D'Arcy et al., 2013). However, biofilms are often very complex in nature. Heterogeneity of natural substrates induces heterogeneous biofilm development that is difficult to measure and observe (Claret, 1998). Devices for sampling deep-sea biofilms are generally lacking, making their study challenging. To avoid these problems, one approach to studying the ecological roles of biofilms is to use artificial substrates to develop biofilms under steady-state conditions. This method has been used in previous studies of biofilm development in hydrothermal vent areas (Guezennec et al., 1998), estuary systems (Jones et al., 2007), and intertidal zones (Salta et al., 2013; Zhang et al., 2013).

Our previous investigation on the metagenomics of biofilms developed on artificial surfaces (Zhang et al., 2014, 2015) has illustrated the developmental processes of biofilms in a brine pool associated with the Thuwal cold seeps (Batang et al., 2012) on the floor of the Red Sea. This brine pool is located at a depth of approximately 850 m and is characterized by a high concentration of potential electron acceptors such as nitrate (18.5 mg L^−1^) and sulfate (2.7 g L^−1^). The particulate organic carbon (POC) concentration was 3.5 mg L^−1^ which was 10 times greater than that of the adjacent normal deep-sea water (NDW) (Zhang et al., 2014, 2015). In contrast, methane and hydrogen sulfide were depleted with respect to NDW. Metagenomics were performed for biofilms developed on three different types of substrates: aluminum (Al), titanium (Ti) and polyvinyl chloride (PVC). Comparisons between brine and NDW biofilms indicated that polysaccharide metabolism related genes were highly enriched in the brine biofilms (Zhang et al., 2015). We also reconstructed two dominant bacterial genomes from the brine biofilm metagenomics datasets, a novel deltaproteobacterium and a novel epsilonproteobacterium. The former metagenome-assembled genome (MAG) possessed enhanced polysaccharide fermentation pathways, whereas the later possessed complete gene clusters for nitrate reduction and nitrogen fixation. We proposed that polysaccharide fermentation and proteolysis interacted with nitrogen cycling to form a complex chain for energy generation in these biofilms (Zhang et al., 2015).

Although, the biofilm development process in the Thuwal cold seep brine pool and the genomics of the two dominant microorganisms has been analyzed, the lifestyle of the other microorganisms in the brine biofilms remains unclear. In the present study, using the genome binning technique, we analyzed 51 partial to near-complete microbial genomes reconstructed from the Al and PVC biofilm metagenomes and the metatranscriptomes derived from these biofilms, toward a systematic understanding of microbial lifestyles in this ecosystem.

## Materials and methods

### Biofilm sampling, DNA extraction and metagenomic analysis

Biofilm sampling was conducted in May 2013 in the Thuwal seeps II (22°16N-38°53E) by the remotely operated underwater vehicle (ROV) Max Rover, DSSI, USA during the Red Sea exploration cruise. Biofilms were developed on six different types of materials in the brine pool for 3 and 6 days. The devices for biofilm development has been reported in Zhang et al. (2014) and the schematic diagrams are also shown in Figure S1 in the present study. The materials were deployed into the brine pool by the ROV. Environmental parameters, such as temperature, salinity, and dissolved oxygen (DO) were measured using conductivity-temperature-depth (CTD) and SBE43 DO sensors attached to the ROV.

The biofilms used in the present study were developed on aluminum and PVC in the brine pool for 6 days and were referred to as “biofilm_Al and biofilm_PVC.” Biofilms on these two materials were selected because biofilm_Al had the highest biomass and PVC represented a biofilm developed on plastic materials. Cells were recovered from the substrates using sterile cell scrapers and suspended in Tris-HCl buffer before extracting nucleic acid using the AllPrep DNA/RNA Mini Kit (Qiagen, Hilden, Germany). The extracted DNA was sequenced using an Illumina HiSeq 2000 platform. Metagenomic datasets of biofilm_Al and biofilm_PVC have been documented in Zhang et al. (2015), and the information of these two metagenomes are also listed in Table S2 in the present study. Assembly of the combined metagenomes of biofilm_Al and biofilm_PVC was performed using SPAdes Genome Assembler 3.6.1 (Bankevich et al., 2012). The specified K values for assembly included 21, 31, 41, 51, 61, 71, and 81, and the “—careful” and “—pe” options were used. The metagenome-based taxonomic structures were obtained by ribosomal database project (RDP) classification (Wang et al., 2007) of the extracted 16S rRNA sequences, and the coverage information.

### Genome binning and validation

Draft genome binning was conducted according to previously described methods (Albertsen et al., 2013; Tian et al., 2014). The genomes were separated mainly on the genome coverage, GC content, and tetranucleotide frequency. The Illumina reads were mapped to the contigs using Bowtie2 (version 2.0.0; Langmead and Salzberg, 2012). The genome coverage was calculated using SAMtools on a local server. The contigs were searched against a set of single-copy protein-encoding genes (Albertsen et al., 2013) using default cut-off values. The single-copy protein-encoding genes identified were searched against the National Center for Biotechnology Information non-redundant (NCBInr) database with BLASTP (e-value < 1e-07) with “xml” output format. Taxonomic information for the contigs was obtained by importing the BLAST results into MEGAN 5.0 (Huson et al., 2011). The metagenome pairs biofilm_Al and biofilm_PVC were selected to obtain the most effective separation and extraction of contigs. It must be noted that the diversity of the biofilm communities was not high and that many contigs belonging to different genomes formed clear, separate clusters based on coverage, facilitating genome binning and elimination of contamination (Figure S2).

Potential contig contamination was examined using CheckM (Parks et al., 2015), and PhylopythiaS, which is a sequence composition-based classifier that utilizes the hierarchical relationships between clades (Patil et al., 2012). Moreover, the Thaumarchaeota MAGs in the present study were compared with the eight genomes obtained using single-cell genomics by Ngugi et al. (2015), based on BLASTP (e-value < 1e-07) and BLASTN (e-value < 1e-07) with predicted open reading frames (ORFs) as input. The results were visualized using the Artemis Comparison Tool (ACT; Carver et al., 2005).

### Genome annotation

The MAGs were annotated by searching the Carbohydrate-Active EnZymes (CAZys) (Cantarel et al., 2009), Kyoto Encyclopedia of Genes and Genomes (KEGG) (Kanehisa and Goto, 2000), and Clusters of Orthologous Groups (COGs) (Galperin et al., 2014) databases. The ORFs in the MAGs were predicted using Prodigal (Hyatt et al., 2010) using a local server. Genes were annotated by BLASTP searches against the above-listed databases using an e-value cut-off of 1e-07 on a local server. The online tool KAAS (KEGG Automatic Annotation Server) was also used to validate the KEGG annotation by local BLAST. Metabolic pathways were analyzed using online tools in KEGGMAPPER (http://www.genome.jp/kegg/mapper.html). Key enzymes revealed by KEGGMAPPER were rechecked against the NCBInr database based on the BLAST results.

### Phylogenetic tree construction

In the phylogenetic analysis of the functional genes (e.g., polysaccharide deacetylase), the protein sequence alignment was determined by the molecular evolutionary genetics analysis using Muscle (MEGA 6.06; Tamura et al., 2013) with the following parameters: gap open -50, cluster method UPGMB and min diagonal length 24. Gblock software (Castresana, 2000) was applied to eliminate less informative sites in the alignments. The construction of maximum-likelihood trees was conducted using MEGA 6.06 with the Tamura-Nei model, the Nearest-Neighbor-Interchange (NNI) method and 500 bootstrap replicates.

### RNA extraction, cDNA amplification and metatranscriptomic sequencing

Biofilms on different substrates were harvested (10 slides per sample) onboard immediately after recovery using sterile cotton tips and stored in RNAlater buffer. RNA extraction was performed using the AllPrep DNA/RNA Mini Kit (Qiagen, Hilden, Germany). Bacterial cells in RNAlater buffer were pelleted by centrifugation at 4000 g for 10 min and then lysed with lysozyme, proteinase K and RLT buffer provided in the kit. Total nucleic acid was extracted, and DNA was removed using the DNA column provided with the AllPrep DNA/RNA Mini Kit. The integrity and quantity of the RNA were checked by agarose gel electrophoresis and a Nanodrop device (ND-1000 spectrophotometer, DiaMed China Limited, Hong Kong), respectively. Prior to first-strand cDNA synthesis, genomic DNA contamination was further removed using the DNA-free™ DNA Removal Kit (Ambion, Austin, TX). The extracted RNA was then used as a template for whole-cDNA amplification using the Ovation Single-Cell RNA-Seq System, which enriches for non-rRNA during cDNA synthesis using proprietary whole transcriptome primers targeting non-rRNA sequences in the transcriptome. The synthesized cDNA was sequenced using an Illumina HiSeq 2000 platform.

### Metatranscriptomic analysis

The information for metatranscriptomic sequences are summarized in Table S2. The sequenced cDNA was subjected to quality control using the next-generation sequencing (NGS) QC toolkit (Patel and Jain, 2012). Assembly of the metatranscriptomic reads was performed using SPAdes Genome Assembler 3.6.1 (Bankevich et al., 2012) on a local server. The specified K values 21, 31, 41, 51, 61, 71, and 81 were used under the “—careful” and “—pe” options. The ORFs were predicted following the steps mentioned above and annotated by BLASTP against the COG -database. To depict the gene expression profiling for individual microorganisms, the reads were aligned to contigs of the MAGs using Bowtie2 (version 2.0.0; Langmead and Salzberg, 2012). Gene expression abundance is the number of transcripts mapped to the CAZys of each MAG that normalized to the length of the genes.

### Data accessibility

The MAGs of Cloacimonetes were deposited in the Whole Genome Shotgun (WGS) database under accession numbers of SAMN04423165, SAMN04423166, SAMN04423167, and SAMN04423168. The MAGs of Marinimicrobia were deposited in the WGS database under accession numbers of SAMN04423157, SAMN04423158, SAMN04423159, SAMN04423160, SAMN04423161, SAMN04423162, and SAMN04423164. The MAG of Bathyarchaeota was deposited in the WGS database under accession number of SAMN04423169.

## Results

### Genome information

Genome binning using two metagenomes, biofilm_Al and biofilm_PVC, generated 51 MAGs of the biofilm-inhabited microorganisms (TCS1-51; “TCS” served as the abbreviation for “Thuwal cold seep”), including members of Actinobacteria (two MAGs belonging to *Candidatus* Microthrix), *Gammaproteobacteria, Deltaproteobacteria, Epsilonprtoteobacteria, Candidatus* Marinimicrobia, *Candidatus* Cloacimonetes, Bathyarchaeota (previously referred to as Miscellaneous Crenarchaeotic Group, MCG) and Thaumarchaeota (Table 1). Based on the metagenome-derived 16S rRNA genes, these phyla were the dominant microbial groups in the biofilms (Figure S3). According to the results of CheckM, these MAGs had a maximum completeness value of 100% and a maximum potential contamination of 2.4%. Based on the results of PhylopythiaS, contigs of most of the MAGs were very consistent, as suggested by the percentage of contigs assigned to a defined taxonomy. For example, 97.2% of the *Pseudomonas* sp. nov (TCS13) contigs could be assigned to the genus *Pseudomonas*. Moreover, contigs of the newly defined bacterial phyla, were compared with their relatives with complete or single-cell derived genomes to assess the potential contamination. For example, 95.8% of the Thaumarchaeota sp. nov. 1 (TCS50) and 91.3% of the Thaumarchaeota sp. nov. 2 (TCS51) could be assigned to the eight single-cell Thaumarchaeota genomes, which were from another brine pool in the Red Sea (Ngugi et al., 2015). The assigned ORFs were distributed in all of the contigs of the TCS MAGs as exemplified by Thaumarchaeota sp. nov. 1 (Figure S4). These results indicated that the MAGs reconstructed in the present study were of high quality.

**Table 1:** **Information for the 51 genomes binned in this study**.

**No**.	**Name**	**Taxa**	**Completeness (%)**	**Size (Mb)**	**No. of contigs**	**N50 of contigs**	**No. of ORFs**	**Contamination (%)**
TCS1	MedAcidi-G2B sp. nov. 1	Actinobacteria	64.86	1.55	204	8506	1717	1.42
TCS2	MedAcidi-G2B sp. nov. 2	Actinobacteria	43.15	1.24	136	9611	1353	1.28
TCS3	MedAcidi-G2B sp. nov. 3	Actinobacteria	32.05	0.87	101	9171	1007	0.00
TCS4	Actinobacterium sp. nov. 1	Actinobacteria	83.99	1.41	97	19,416	1399	0.43
TCS5	Actinobacterium sp. nov. 2	Actinobacteria	98.26	3.58	32	205,730	3268	0.00
TCS6	Microthrix bacterium sp. nov. 1	Microthrix (Actinobacteria)	31.35	1.46	173	9813	1598	1.72
TCS7	Microthrix bacterium sp. nov. 2	Microthrix (Actinobacteria)	53.76	1.50	205	8324	1648	0.00
TCS8	*Alteromonas* sp. nov.	Gammaproteobacteira	10.34	0.96	152	6351	964	0.00
TCS9	*Marinobacter* sp. nov. 2	Gammaproteobacteira	100.00	4.20	93	79,392	3983	0.50
TCS10	*Marinobacter* sp. nov. 3	Gammaproteobacteira	100.00	4.23	46	213,908	3905	0.50
TCS11	*Oleispira* sp. nov. 1	Gammaproteobacteira	6.04	0.38	32	16,330	377	0.08
TCS12	*Oleispira* sp. nov. 2	Gammaproteobacteira	40.43	1.59	349	5791	1766	0.63
TCS13	*Pseudomonas* sp. nov.	Gammaproteobacteira	44.05	2.72	288	9706	2797	0.34
TCS14	*Simiduia* sp. nov.	Gammaproteobacteira	28.33	1.33	248	5326	1415	0.46
TCS15	*Thiomicrospira* sp. nov.	Gammaproteobacteira	80.29	1.75	192	12,088	1775	0.44
TCS16	*Vibrio* sp. nov	Gammaproteobacteira	28.45	1.51	257	6415	1565	0.00
TCS17	*Neptuniibacter* sp. nov	Gammaproteobacteira	6.90	0.40	3	251,948	367	0.00
TCS18	Gammaproteobacterium sp. nov. 1	Gammaproteobacteira	14.00	0.92	9	162,608	1015	0.00
TCS19	Gammaproteobactirium sp. nov. 2	Gammaproteobacteira	78.85	3.44	174	29,544	3202	1.35
TCS20	Gammaproteobacterium sp. nov. 3	Gammaproteobacteira	74.00	3.07	80	59,528	2849	1.57
TCS21	Gammaproteobacterium sp. nov. 4	Gammaproteobacteira	28.45	2.03	180	11,587	2100	0.00
TCS22	Gammaproteobacterium sp. nov. 5	Gammaproteobacteira	82.42	2.25	223	13,651	2486	1.52
TCS23	Gammaproteobacterium sp. nov. 6	Gammaproteobacteira	39.00	0.43	113	3840	496	0.00
TCS24	Gammaproteobacterium sp. nov. 7	Gammaproteobacteira	61.62	1.44	76	38,495	1539	1.78
TCS25	Gammaproteobacterium sp. nov. 8	Gammaproteobacteira	72.73	1.41	133	14,142	1519	0.00
TCS26	*Desulfobacter* sp. nov.	Deltaproteobacteira	41.38	1.81	242	7562	1966	1.72
TCS27	*Desulfobacterales* sp. nov.	Deltaproteobacteira	66.18	1.70	162	11,898	1757	2.40
TCS28	*Desulfobacula* sp. nov. 1	Deltaproteobacteira	45.13	1.37	243	6245	1474	0.00
TCS29	*Desulfobacula* sp. nov. 2	Deltaproteobacteira	91.61	2.40	183	17,327	2415	0.00
TCS30	*Desulfobacula* sp. nov. 3	Deltaproteobacteira	44.81	1.27	204	6824	1350	0.00
TCS31	*Desulfuromonas* sp. nov.	Deltaproteobacteira	57.42	1.28	92	17,683	1267	0.86
TCS32	Deltaproteobacterium sp. nov. 1	Deltaproteobacteira	89.58	2.74	138	26,077	2527	0.00
TCS33	Deltaproteobacterium sp. nov. 2	Deltaproteobacteira	75.33	2.07	241	9386	2088	0.00
TCS34	Deltaproteobacterium sp. nov. 3	Deltaproteobacteira	98.13	3.14	145	31,122	3075	0.62
TCS35	*Arcobacter* sp. nov. 1	Epsilonproteobacteira	60.70	1.03	162	7068	1226	0.20
TCS36	*Arcobacter* sp. nov. 2	Epsilonproteobacteira	80.49	1.23	123	13,070	1421	0.20
TCS37	Epsilonproteobacterium sp. nov. 3	Epsilonproteobacteira	59.19	0.85	157	5800	900	0.10
TCS38	Marinimicrobia bacterium sp. nov. 1	Marinimicrobia	75.82	1.15	95	15,228	1123	0.00
TCS39	Marinimicrobia bacterium sp. nov. 2	Marinimicrobia	26.72	1.27	138	9804	1312	1.72
TCS40	Marinimicrobia bacterium sp. nov. 3	Marinimicrobia	39.62	1.26	155	10,981	1313	2.40
TCS41	Marinimicrobia bacterium sp. nov. 4	Marinimicrobia	87.91	1.68	98	27,341	1703	0.65
TCS42	Marinimicrobia bacterium sp. nov. 5	Marinimicrobia	92.86	1.91	121	20,471	1815	2.20
TCS43	Marinimicrobia bacterium sp. nov. 6	Marinimicrobia	14.29	0.57	55	11,871	547	1.10
TCS44	Marinimicrobia bacterium sp. nov. 7	Marinimicrobia	14.29	0.41	26	21,486	453	1.10
TCS45	Cloacimonetes bacterium sp. nov. 1	Cloacimonetes	55.13	0.66	126	5368	703	0.10
TCS46	Cloacimonetes bacterium sp. nov. 2	Cloacimonetes	58.09	1.06	196	5684	1079	1.20
TCS47	Cloacimonetes bacterium sp. nov. 3	Cloacimonetes	87.16	1.38	196	7570	1403	0.00
TCS48	Cloacimonetes bacterium sp. nov. 4	Cloacimonetes	36.07	0.57	120	4938	622	1.10
TCS49	Bathyarchaeota sp. nov^*^	Bathyarchaeota	59.35	1.54	137	12,550	1882	2.34
TCS50	Thaumarchaeota sp. nov. 1	Thaumarchaeota	34.30	0.37	42	7826	527	0.00
TCS51	Thaumarchaeota sp. nov. 2	Thaumarchaeota	55.18	0.59	80	8675	796	0.00

The completeness and potential contamination were assessed by CheckM.

* Previously referred to as Miscellaneous Crenarchaeotic Group (MCG).

### Central metabolic pathway

A large proportion of the genes identified in many of the MAGs were found to be involved in fermentative carbohydrate metabolism. The distribution of CAZys (Cantarel et al., 2009) responsible for the degradation of complex carbohydrates is listed in Table S1, such as β-glucosidase (GH3), α-amylase (GH13) and endo-β-1,4-galactanase (GH53). In total 17 unique CAZy categories corresponding to 1692 genes were identified in the 51 MAGs (on average 33 genes/genome). Genes encoding proteins playing a role in electron transport and energy production, including those encoding F_420_H_2_ dehydrogenase (fpo), cytochrome C (cytoC), the rhodobacter nitrogen fixation (rnf) complex, nitrate reductase and sulfate reductase, were identified in many of the MAGs. Annotation by COGs (Galperin et al., 2014) showed a high diversity in the gene arrangement of the F_420_ biosynthesis gene clusters (Figure S5), which were present in eight of the MAGs belonging to different phyla. Moreover, putative bacterial microcompartment (BMC) clusters, types of protein-bound bacterial organelles that function in carbon catabolism and storage (Nobu et al., 2016), were present in five MAGs including those of Actinobacteria, Marinimicrobia and *Desulfobacter* (Figure S6). As reported by Nobu et al. (2016), genes related to aldehyde and sugar metabolism are present in the BMC clusters, including rnf-C like NADH dehydrogenase (COG4656), sugar isomerase (COG0363) and aldehyde dehydrogenase (COG1012). However, other genes were also identified in the present study, such as NTP pyrophosphohydrolases and oxidative damage repair enzymes (COG0494), making the BMC clusters different from those in previous study.

**Figure 1:**
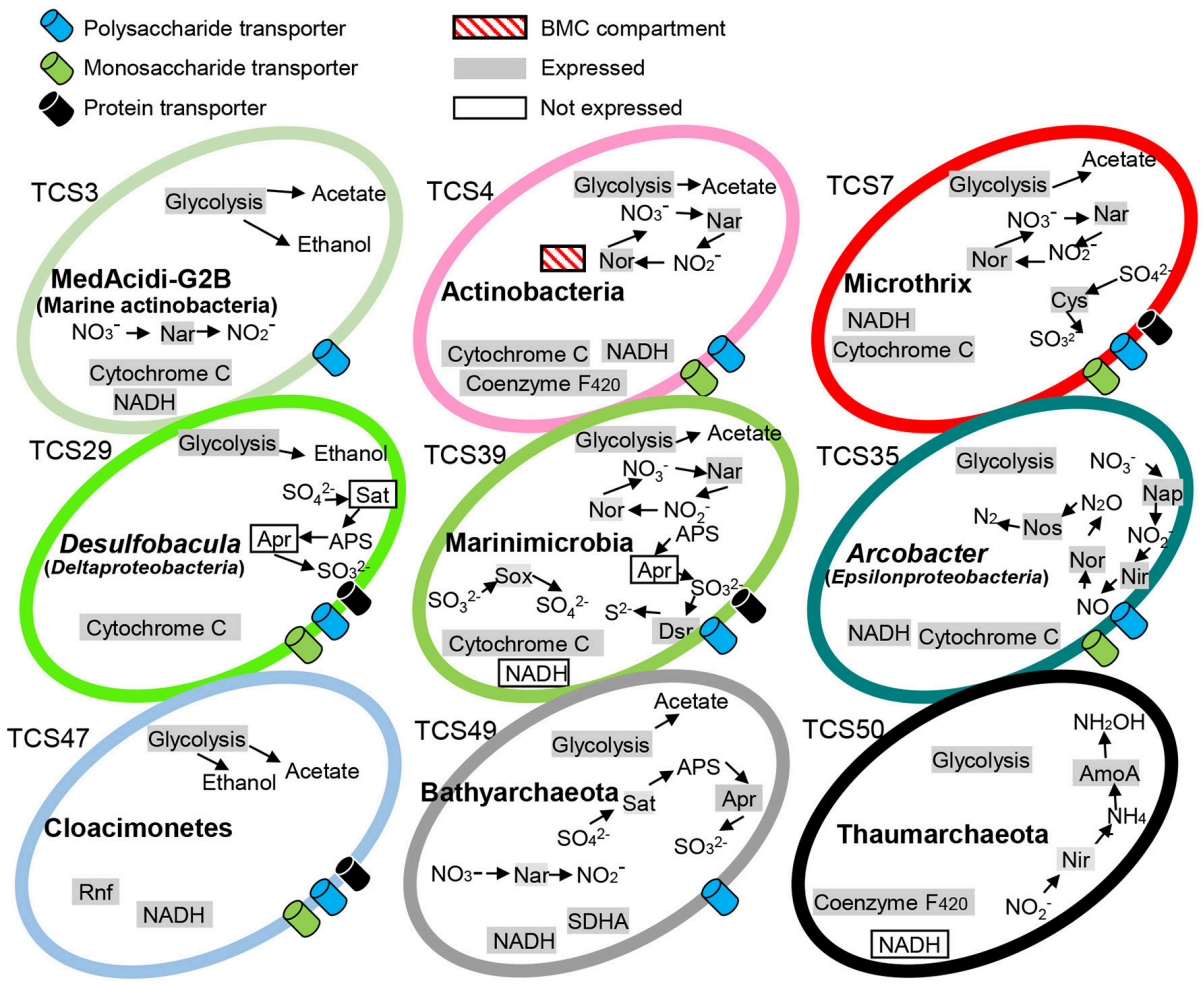
**Metabolic capacities of representative microorganisms based on genomic and metatranscriptomic analyses**. The complete pathways responsible for carbohydrate fermentative metabolism as well as energy generation were detected in the MAGs. The expression profiles of these pathways were examined by mapping transcripts to key functional genes. TCS, Thuwal cold seep; Rnf, Rhodobacter nitrogen fixation; BMC, putative bacterial microcompartment; SDHA, succinate dehydrogenase complex subunit A. The 51 MAGs were numbered TCS1-51.

The prevalence and versatility of carbohydrate metabolism and energy generation was further supported by KEGG-based pathway reconstruction. The central metabolic strategies are summarized in Figure 1. Many of the MAGs encoded proteins associated with monosaccharide and polysaccharide transport, nitrate and sulfate reduction, electron transport systems (ETSs), glycolysis, and reoxidation of energy-conserving elements via acetyl-CoA reduction to ethanol or acetate. For example, monosaccharide and polysaccharide transport was indicated by the presence of a number of ABC transporters in all the bacterial genomes; dissimilatory nitrate reduction was indicated by both respiratory nitrate reductase (*nar*) and periplasmic nitrate reductases (*nap*); dissimilatory sulfate reduction was indicated by sulfur oxidation (SOX) gene cluster and adenylylsulfate reductase; and acetate generation was indicated by acetate kinase. Notably, the Marinimicrobia bacterium sp. nov. 2 (TCS39) and Bathyarchaeota sp. nov (TCS49) possessed both dissimilatory nitrogen and sulfur reduction pathways.

### Unique genomic features

When the TCS MAGs were compared with reference genomes within the same phylum, unique features concerning carbohydrate metabolism and energy generation were observed. For example, genes encoding NADH dehydrogenase, such as *nuoB, nuoL, ndufv2, NdhJ*, and *NoxF* were only identified in four Cloacimonetes MAGs (TCS45-47) when compared with complete or single-cell Cloacimonetes genomes (Figure S7); similar results were observed for chitin deacetylase (EC3.5.1.41) and the α-D-glucose transformation pathway, which are important steps in glycolysis. Moreover, the complete pyruvate to acetate pathway was identified in Marinimicrobia bacterium sp. nov. 2 (TCS39) and Marinimicrobia bacterium sp. nov. 3 (TCS40), but the enzymes included in this pathway could not be found in the four reference genomes, the single-cell Marinimicrobia genomes from previous studies (Rinke et al., 2013; Nobu et al., 2015) (Figure S8). In addition, the Bathyarchaeota MAG (TCS49) harbored specific genes for chitin, cellulose, and β-D-glucose metabolism, such as a glycoside hydrolase family 3 gene (probably chitin deacetylase; EC3.5.1.41) and β-glucosidase (EC3.2.1.4) (Figure S9); the presence of *aprAB* and *cysC* genes, which are involved in dissimilatory sulfate reduction, differentiated TCS49 from the reference genomes.

**Figure 2:**
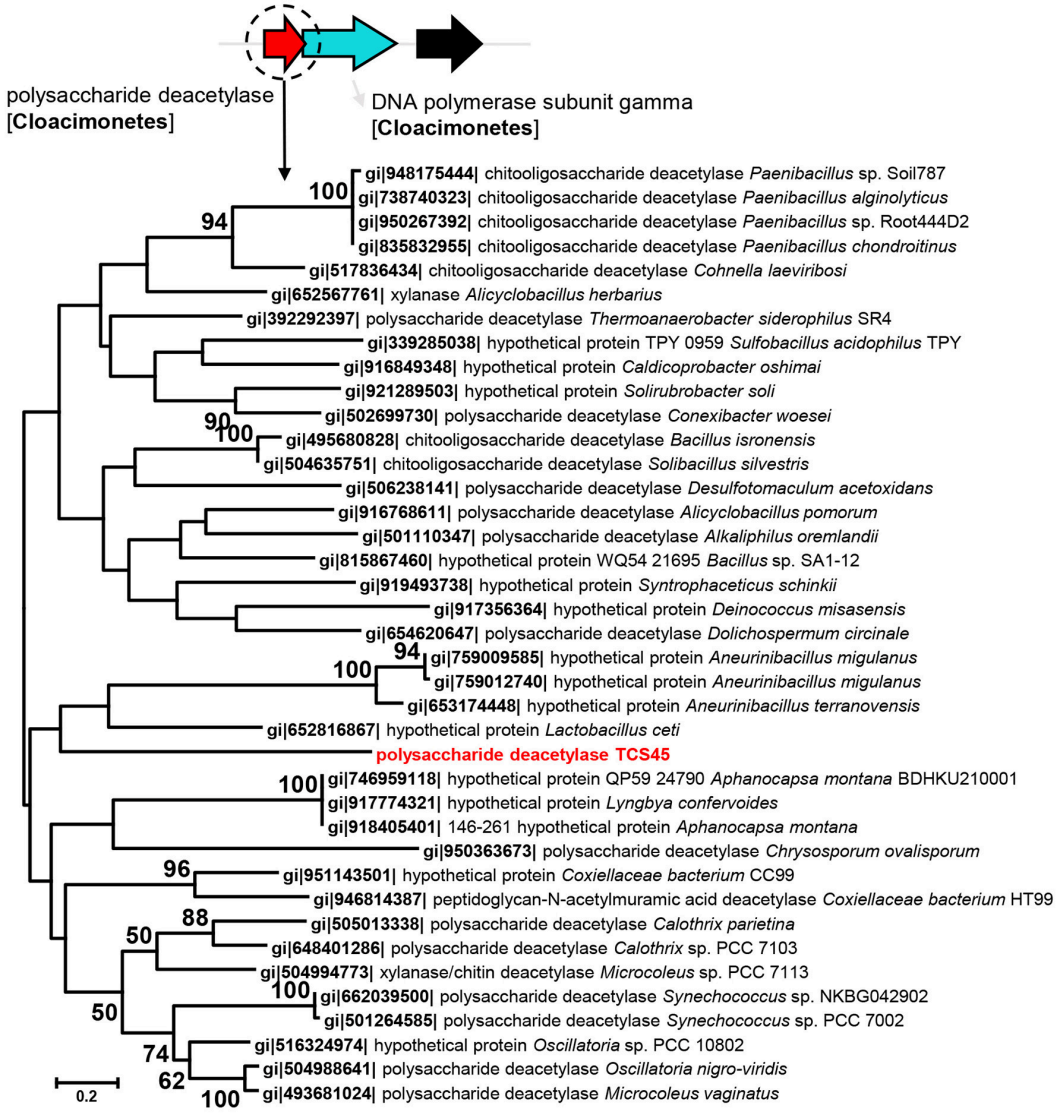
**Maximum likelihood phylogenetic tree of the polysaccharide deacetylase in Cloacimonetes bacterium sp. nov. 1**. The reference sequences were obtained from NCBI databases. Bootstrap values based on 500 replicates are shown at the nodes. The taxonomic affiliation of the adjacent genes, based on the best hits in the BLASTP search against the NCBInr database are also shown to eliminate the possibility of contig contamination.

Given that a number of unique genes were identified from these MAGs as compared with their relatives, we examined the phylogeny of these genes. The polysaccharide deacetylase (EC3.2.1.21) in Cloacimonetes bacterium sp. nov. 1 (TCS45) was phylogenetically close to the polysaccharide deacetylase from *Lactobacillus ceti* (Figure 2). Although, the bootstrap values in the tree were not high, BLAST analysis also revealed close relationship between polysaccharide deacetylase in Cloacimonetes bacterium sp. nov. 1 and those from firmicutes. The nitrate reductase α and β units in Marinimicrobia bacterium sp. nov. 3 (TCS40) were phylogenetically close to the nitrate reductases from *Geothrix fermentans* (Figures S10, S11). In addition, the glycoside hydrolase family 3 gene in Bathyarchaeota sp. nov (TCS49) was affiliated with *Hungatella hathewayi* and *Clostridium hathewayi* (Figure S12). The adjacent genes in the same contigs, which were affiliated with Cloacimonetes, Marinimicrobia or Bathyarchaeota, were displayed to confirm that the presence of these novel genes was probably not attributable to contig contamination.

### Genome-wide expression profiling

**Figure 3:**
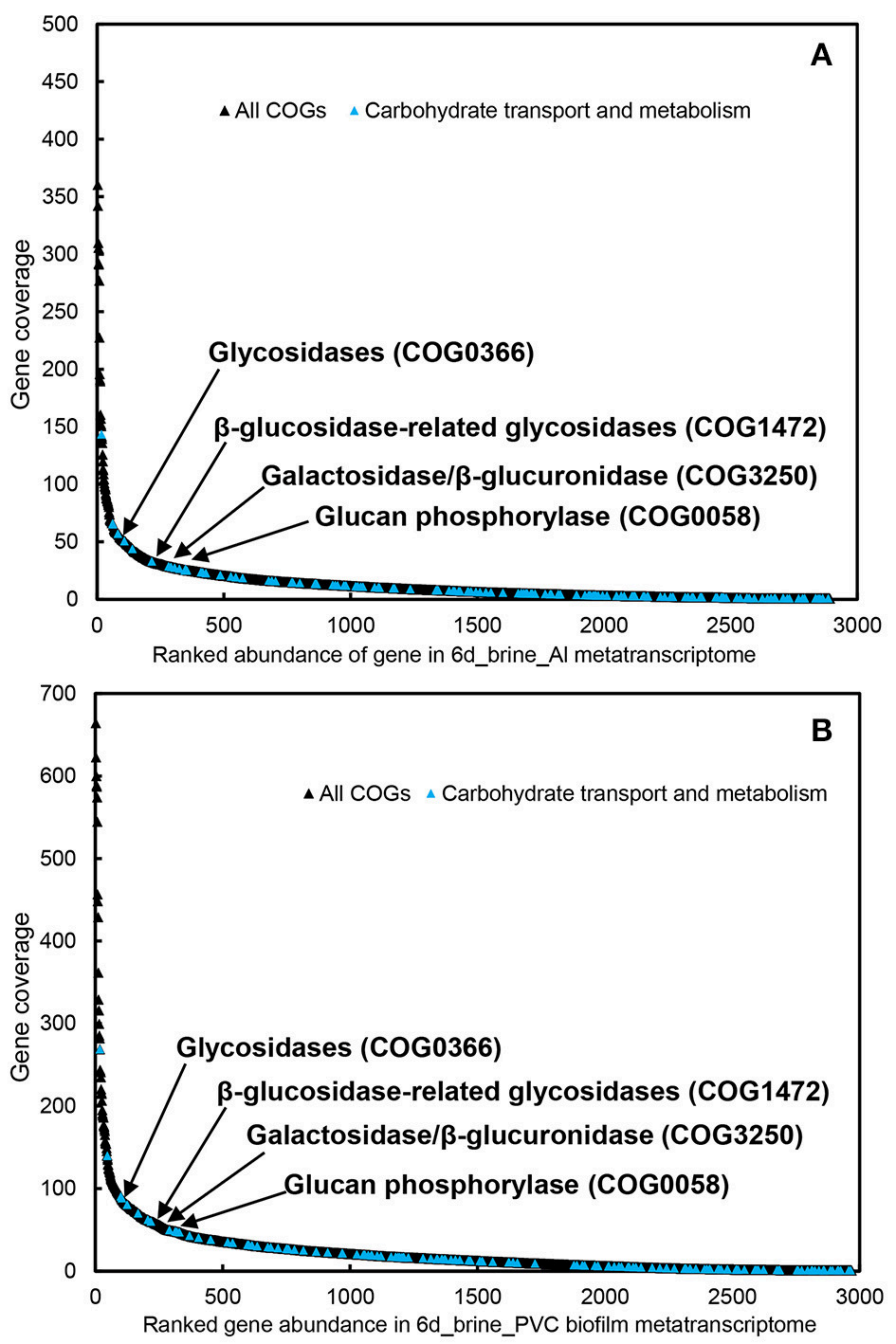
**Transcript abundance of all COGs and carbohydrate transport and metabolism genes in the two biofilm metatranscriptomes** (**A**, biofilm_Al; **B**, biofilm_PVC). Genes for Glycosidases (COG0366), β-glucosidase-related glycosidases (COG1472), galactosidase/β-glucuronidase (COG3250), and glucan phosphorylase (COG0058) are highlighted in the figure.

To explore the major active functions, we obtained ~600 Mb of metatranscriptomic datasets for the biofilms developed on Al and PVC. The metatranscriptomic sequences were searched against the COG database, revealing an overall picture of the active functions. Transcripts of the genes involved in carbohydrate transport and metabolism were among the most abundant in the metatranscriptomes, particularly the glycosidases (COG0366), β-glucosidase-related glycosidases (COG1472) and β -galactosidase/beta-glucuronidase (COG3250) (Figure 3). In contrast, few peptidases that are essential for protein metabolism were identified, and rank analysis suggested relative lower expression levels for these genes (Figure S13).

**Figure 4:**
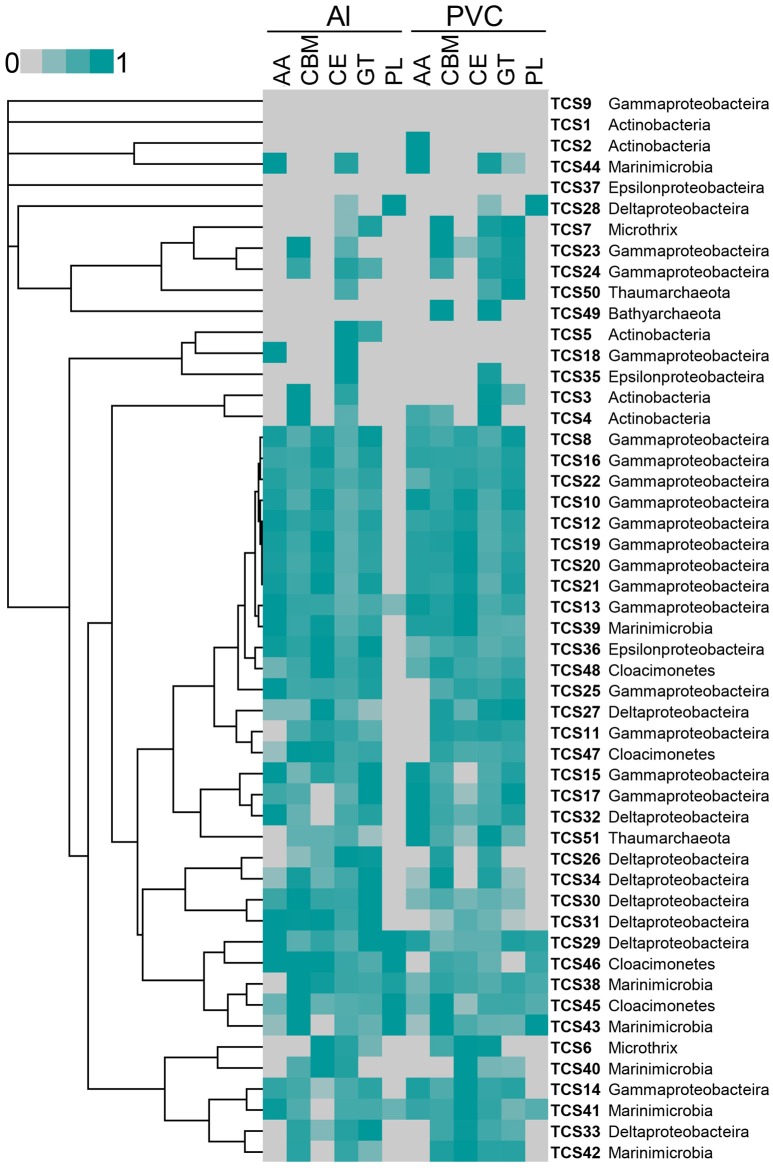
**Relative abundance of expressed genes for carbohydrate metabolism from each MAG**. Expression levels for auxiliary activities (AAs), carbohydrate-binding module (CBMs), carbohydrate esterases (CEs), glycoside hydrolases (GHs), Glycosyltransferase (GTs), and polysaccharide lyases (PLs) from each MAG in the biofilm_Al and biofilm_PVC metatranscriptomes are shown. Gene expression abundance is the number of transcripts mapped to the CAZys of each MAG that normalized to the length of the genes. The highest expression level was normalized as “1.”

The reads from these two metatranscriptomes were mapped to the 51 MAGs. Abundant active bacteria could be identified for nearly all of the phyla, whereas rare active bacteria were also included in each phylum. Key functional genes involved in nitrate and sulfate respiration were expressed, such as the *napAB, narGH, nirK, norC, nosZ*, and sulfur oxidation (SOX) gene cluster. The gene expression information was integrated in to pathways shown in Figure 1. Moreover, when the metatranscriptomic sequences were mapped to CAZys in each MAG, a heatmap showing expression pattern of CAZys was generated (Figure 4). The results confirmed the expression of diverse carbohydrate metabolism related genes, and indicated relative higher expression of the glycoside hydrolases (GHs), glycosyltransferase (GTs) than enzymes in other families.

## Discussion

We reconstructed 51 partial to nearly complete MAGs with the aim of investigating the microbial lifestyles in brine pool biofilm communities. We validated the genome binning results using different approaches before the subsequent analyses. Genomic and transcriptomic analyses provided evidence that organic carbon metabolism utilizing nitrate, nitrite, and sulfate as potential electron acceptors is prevalent among these microorganisms. In particular, we identified several unique metabolic pathways for Cloacimonetes, Marinimicrobia, and Bathyarchaeota, which differ from previously reported lifestyles for these microbial groups.

It is important to validate the results from genome binning, although this approach has been repeatedly employed in various studies (Brown et al., 2015; Emerson et al., 2016). We suggest that validation using PhylopythiaS is suitable for genome bins of well-documented microbial taxa, such as *Pseudomonas*. But for novel phyla without many available genomes in public databases, comparing to single-cell amplified genomes (SAGs) from similar habitats is a more effective approach. The Thaumarchaeota genome binned in the present study possesses a gene inventory that is nearly identical to the SAGs from another Red Sea brine pool (Ngugi et al., 2015); this is strong evidence that the genome binning approach employed here is highly accurate.

The overall profiling of the genomics suggested the prevalence of organic carbon metabolism using diverse electron transfer elements. This notion is supported by the high diversity or specificity of particular pathways. Previous studies have discussed the distribution of F_420_ biosynthesis genes in the genomes of microorganisms other than Archaea and Actinobacteria (Selengut and Haft, 2010; Li et al., 2014). In the present study, we showed a higher diversity of the F_420_ biosynthesis gene cluster than previously assumed, suggesting a possible correlation between these genes and microbial adaptation to the brine pool. The presence of BMC compartments provides additional evidence that carbohydrate metabolism is particularly important for microbial diversity in this habitat, consistent with the concept that carbon storage is a mechanism conferring microbial resistance to hypersaline stresses (Werner et al., 2014). Notably, environmental adaptation of the Thuwal cold seep brine residents seems to have been facilitated by horizontal gene transfer (HGT) from various bacteria, as indicated by the phylogeny of several novel genes in the genome bins. The metatranscriptomic results suggested that anaerobic carbohydrate catabolism modulates not only genomic content but also post-genomic activity.

With respect to the detailed microbial lifestyles, this study is the first to report the presence of certain fermentative groups in a brine environment. To date, Microthrix have only been recorded in activated sludge ecosystems (Muller et al., 2012; McIlroy et al., 2013); Actinobacterium MedAcidi-G2B, for which little genomic information is available, have been reported only in the deep-sea habitat of the Mediterranean Sea (Mizuno et al., 2015); and Cloacimonetes are usually found in wastewater treatment plants or bioreactors (Pelletier et al., 2008; Nobu et al., 2015). Cloacimonetes and Marinimicrobia were found to be proteolytic amino-acid degraders and syntrophic propionate degraders, respectively (Nobu et al., 2015). However, the genomic and metatranscriptomic analyses of the present study showed their capacity to perform carbohydrate fermentation. The Bathyarchaeota may also be able to degrade polysaccharides, as suggested by the presence and expression of unique chitinase, cellulase and a rather complete acetate fermentation pathway in the MAG. The Bathyarchaeota MAG harbors a dissimilatory sulfate reduction pathway, differentiating it from other Bathyarchaeota members (Lloyd et al., 2013; Meng et al., 2014; Fillol et al., 2016) and suggesting the metabolic plasticity of this archaeal group. Nitrate or sulfate reducers, such as unclassified members of the *Deltaproteobacteria* and *Epsilonproteobacteria*, are also probable important players in polysaccharide fermentation, supporting the metabolic interconnection of carbohydrate metabolism with sulfur and nitrogen cycles in the brine pool.

## Conclusion

Microbial consumption of organic carbons that sink from the surface ocean to deep-sea environments contributes to the remineralization of so called “dead organic matter” or “fecal material.” Although, organic carbon consumption by deep-sea bacteria and archaea has not been quantified, our findings demonstrated the participation of diverse and ubiquitous microbial taxa in this process. The niche-specific functions suggest a long history of microbial adaptation to the brine pool, where anaerobic organic carbon metabolism is likely to be an important driving force. Our findings shed new light on the roles of microorganisms in carbon cycling in deep-sea environments.

## Author contributions

PQ: Designed the study; WZ, WD, BY, RT, SG, and HL: Performed the analyses. All authors wrote the manuscript and the Supplementary Materials.

### Conflict of interest statement

The authors declare that the research was conducted in the absence of any commercial or financial relationships that could be construed as a potential conflict of interest.
