# Supplementary material for: Genomic and Transcriptomic Evidence for Carbohydrate Consumption among Microorganisms in a Cold Seep Brine Pool
Source: Front Microbiol. 2016 Nov 15;7:1825. doi: 10.3389/fmicb.2016.01825 (PMC5108811; doi:10.3389/fmicb.2016.01825)
Supplement: Supplementary file 1 [file DataSheet1.doc]

**Supplementary Materials**

**Genomic and Transcriptomic Evidence for Carbohydrate Consumption Among Microorganisms in a Cold Seep Brine Pool**

Weipeng Zhang^1*^, Wei Ding^1*^, Bo Yang^1^, Renmao Tian^1^, Shuo Gu^1^, Haiwei Luo^2^, and Pei-Yuan Qian^1#^

^1^Division of Life Science, Hong Kong University of Science and Technology, Clear Water Bay, Hong Kong;

^2^Simon F. S. Li Marine Science Laboratory, School of Life Sciences, The Chinese University of Hong Kong, Shatin, Hong Kong.

**Running title:** Genomics of deep-sea microorganisms

**Keywords:** brine pool, biofilm, carbon metabolism, microbial genomics, transcriptomics

^*^These authors contributed equally to this work

^#^ Corresponding author:

Pei-Yuan Qian, PhD, Chair professor, Division of Life Science,

The Hong Kong University of Science and Technology, Clear Water Bay, Hong Kong

Phone: 0852-2358-7331

Fax: 0852-2358-1559

E-mail: boqianpy@ust.hk


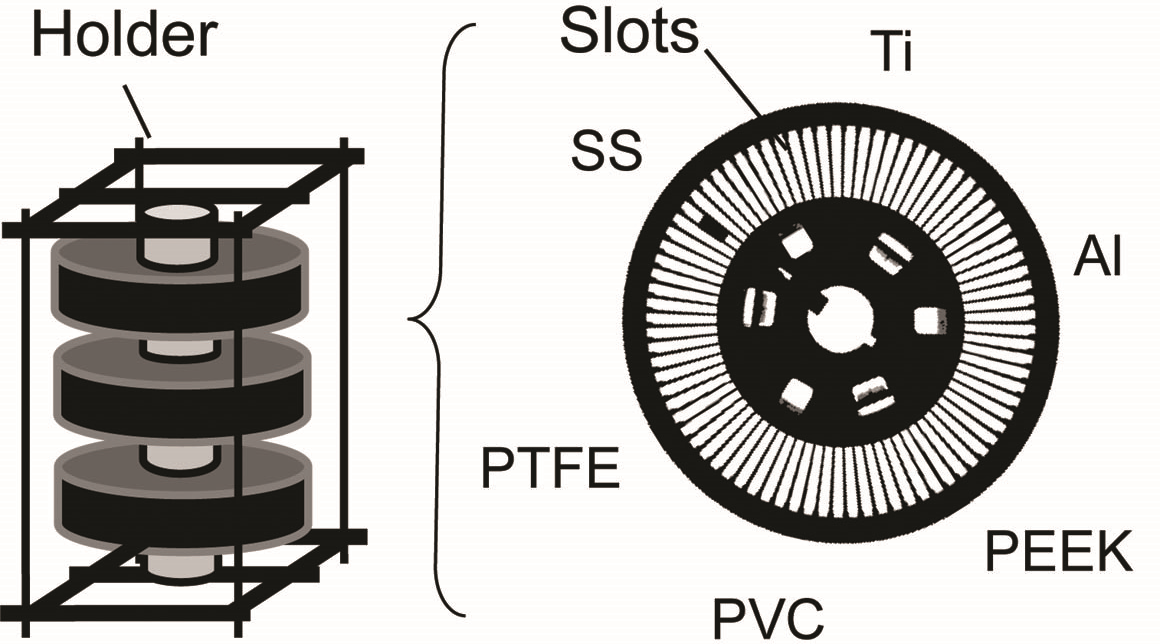


**Figure S1.** Devices used for biofilm development in the Thuwal cold seeps II brine pool. The samplers contained six types of substrate, including aluminum (Al), polyether ether ketone (PEEK), Polyvinyl chloride (PVC), polytetrafluoroethene (PTFE), stainless steel (SS) and titanium (Ti). Material pieces could be fixed into the slots of the carousel.


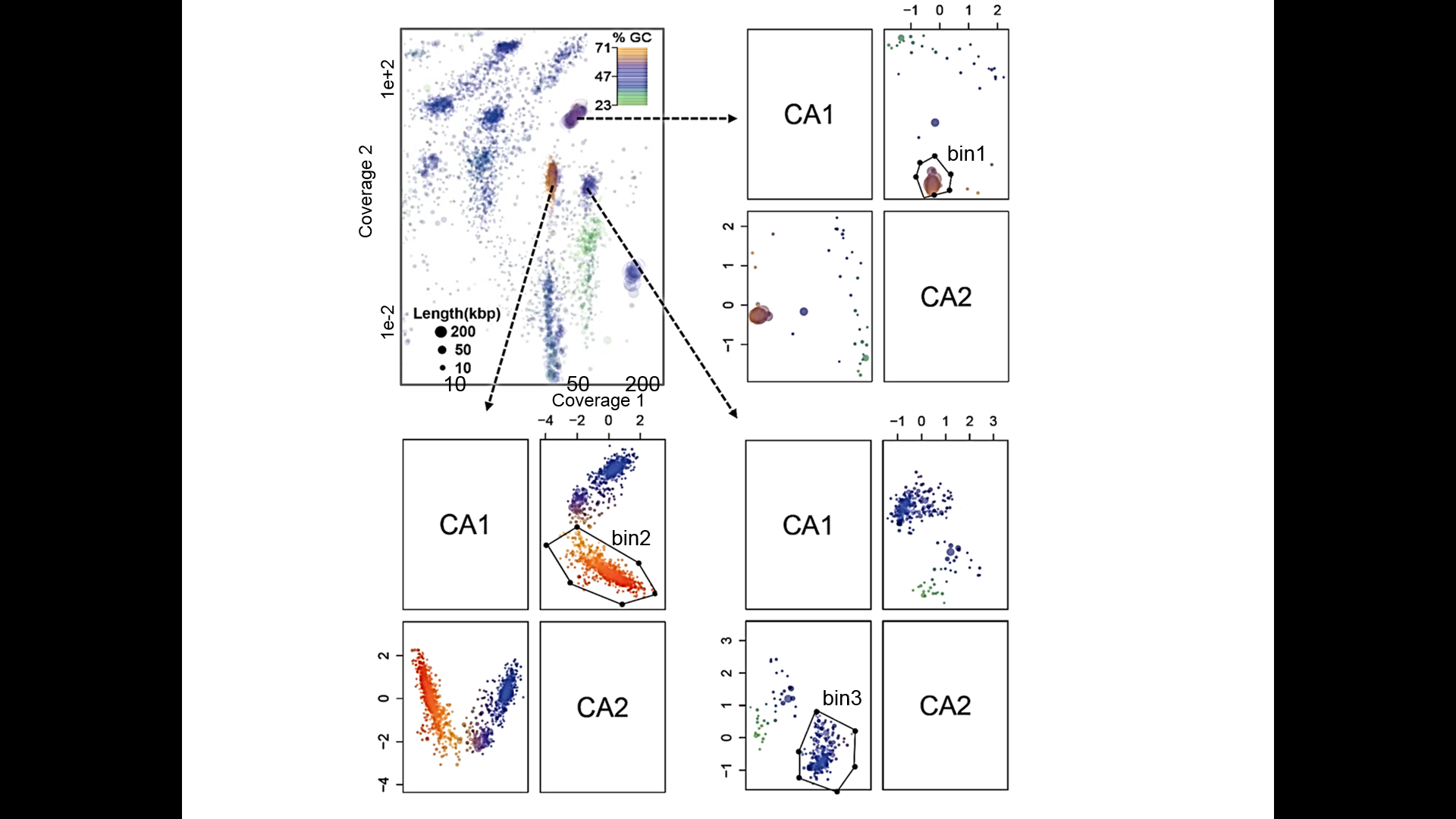


**Figure S2.** Representative figures showing the procedures of genome binning. Extraction of three genome bins are shown. Each circle represents a metagenomic contig with size proportional to the length and colored by GC content while only contigs >1000 bp were used. Cluster analysis (CA) based on tetranucleotide frequencies was performed for further binning.

**Figure S3.** Taxonomic classification of qualified microbial reads retrieved from the two biofilm metagenomes in the brine pool. Sequences were classified to the phylum level (Class level for Proteobacteria) using the RDP classifier.

**Figure S4.** Sequence comparison between Thaumarchaeota sp. nov. 1 (TCS65) and the single-cell genomes from another brine pool in the Red Sea (Ngugi et al*.*, 2015). Comparison was performed based on BLASTN (e-value <1e-07) using predicted ORFs as input, and then visualized using Artemis Comparison Tool (ACT). Only hits with an e-value of 0 are shown, and these hits are distributed in all the contigs.

**Figure S5.** Distribution of F_420_ biosynthesis genes in eight of the genome bins. The 2-phospho-L-lactate transferase *cofD* is present in all clusters. Contig contamination was identified through BLASTX search in the NCBInr database using adjacent genes as queries.

**Figure S6.** Distribution of the putative bacterial compartment (BMC) biosynthesis genes in five of the genome bins. COGs 4576 and 4577 are the conserved shell proteins of the BMC. Contig contamination was identified by BLASTX search in the NCBI-Nr database using adjacent genes as queries.

**Figure S7.** KEGG pathways differentiate Cloacimonetes MAGs (TCS45-47) and the reference Marinimicrobia genomes. The complete genome Cloacamonas acidaminovorans str. Evry (Pelletier et al., 2008) and single-cell genomes Cloacimonetes bacterium JGI OUT-1, Cloacimonetes bacterium JGI OUT-2, Cloacimonetes bacterium JGI OUT-3 and Cloacimonetes bacterium JGI 0000014-k11 (Rinke et al., 2013; Nobu et al*.*, 2015) were used for comparison. Enzyme commission number or gene names are labeled.

**Figure S8.** KEGG pathways differentiate Marinimicrobia MAGs (TCS38-41) and the reference Marinimicrobia genomes. The single-cell genomes Marinimicrobia bacterium JGI OTU-1, Marinimicrobia bacterium JGI 0000113-D11, Marinimicrobia bacterium JGI 0000059-E23, and Marinimicrobia bacterium SCGC AAA003-L08 (Rinke et al., 2013; Nobu et al*.*, 2015) were used for comparison. Enzyme commission number or gene names are labeled.

**Figure S9.** KEGG pathways differentiate the MAG of Bathyarchaeota (TCS49) and the reference genomes. One Bathyarchaeota genome bin (Lloyd et al., 2013), two genomic fragments (Meng et al., 2014) and five draft genomes available in NCBI were used for comparison. Enzyme commission number or gene names are labeled.

**Figure S10.** Maximum likelihood phylogenetic tree of the nitrate reductase α-unit (NarG) in Marinimicrobia bacterium sp. nov. 3. The reference sequences were downloaded from NCBI databases. Bootstrap values based on 500 replicates are shown at the nodes. The taxonomic affiliation of the adjacent genes, based on the best hits in the BLASTP search against the single-cell Marinimicrobia genomes are also shown to eliminate the possibility of contig contamination.

**Figure S11.** Maximum likelihood phylogenetic tree of the nitrate reductase β-unit (NarH) in Marinimicrobia bacterium sp. nov. 3. The reference sequences were obtained from NCBI databases. Bootstrap values based on 500 replicates are shown at the nodes. The taxonomic affiliation of the adjacent genes, based on the best hits in the BLASTP search against the single-cell Marinimicrobia genomes are also shown to eliminate the possibility of contig contamination.

**Figure S12.** Evidences for horizontal gene transfer (HGT) of a family 3 glycoside hydrolase. Maximum-likelihood phylogenetic tree of the family 3 glycoside hydrolase in Bathyarchaeota sp. nov is shown. The reference sequences were obtained from NCBI databases. Bootstrap values based on 500 replicates are shown at the nodes. The taxonomic affiliation of the adjacent genes, based on the best hits in the BLASTP search against the NCBI-Nr database, are also shown to eliminate the possibility of contig contamination.


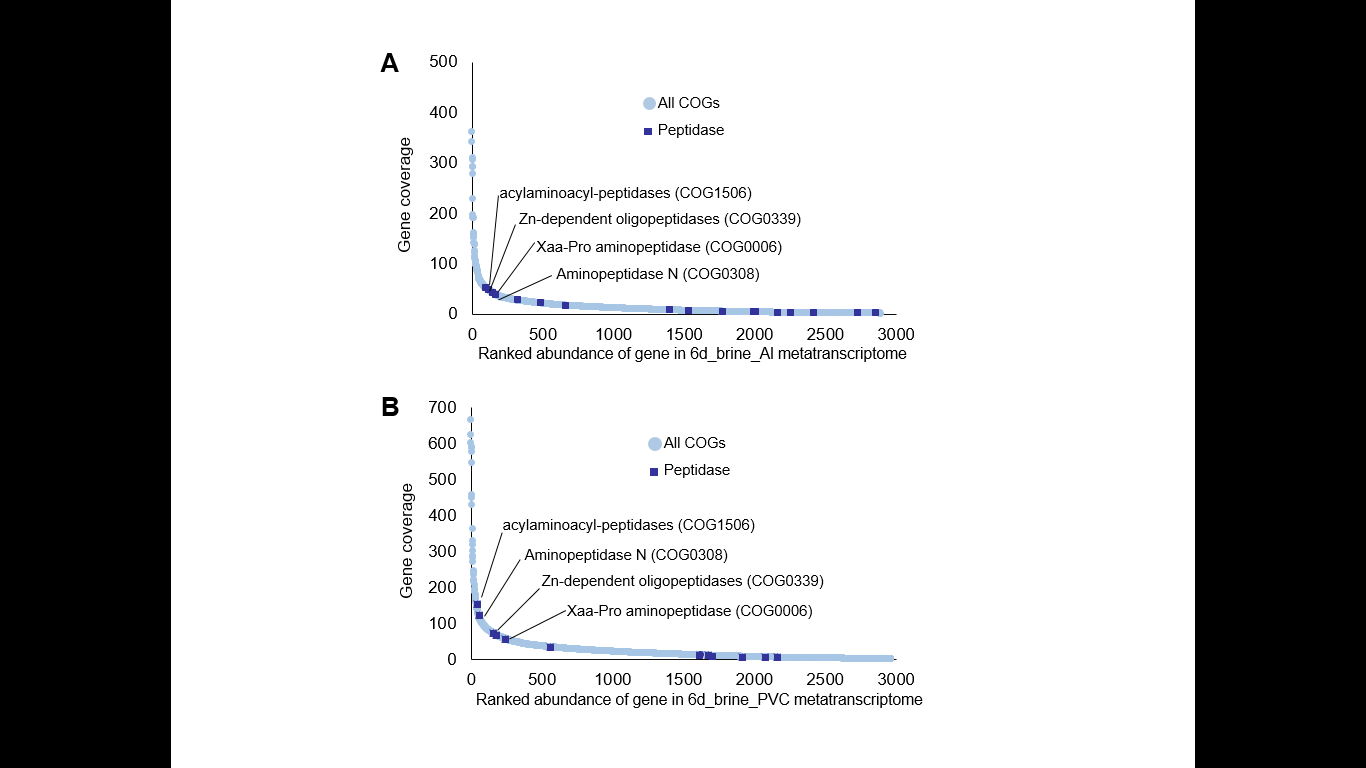


**Figure S13.** Transcript abundance of all COGs and peptidase genes in the biofilm metatranscriptomes.

**Table S1 Distribution of CAZy enzymes in the 51 genome bins.**

| **CAZys** |  | **Genome bins** | | | | | | | | | | | | | | | | | | | | | | | | | |
| --- | --- | --- | --- | --- | --- | --- | --- | --- | --- | --- | --- | --- | --- | --- | --- | --- | --- | --- | --- | --- | --- | --- | --- | --- | --- | --- | --- |
|  |  | **1** | **2** | **3** | **4** | **5** | **6** | **7** | **8** | **9** | **10** | **11** | **12** | **13** | **14** | **15** | **16** | **17** | **18** | **19** | **20** | **21** | **22** | **23** | **24** | **25** | **26** |
| GH1 | β-glucosidase, β-galactosidase and others | 4 | 4 | 1 | 1 | 21 | 11 |  | 6 | 8 | 6 | 8 | 2 | 4 | 5 | 2 | 5 | 9 | 5 | 7 | 6 | 6 | 8 | 11 | 8 | 4 | 9 |
| GH2 | β-galactosidase, β-glucuronidase, β-mannosidase and others | 1 | 1 | 0 |  |  | 1 |  |  |  | 1 |  | 1 | 1 | 1 |  |  | 1 |  |  |  | 1 | 2 | 2 | 2 | 1 | 2 |
| GH3 | β-glucosidase, xylan 1,4-β-xylosidase | 3 | 4 | 4 | 1 | 8 | 5 | 1 | 8 | 3 | 2 | 4 | 2 | 1 | 2 | 4 | 4 | 4 |  | 3 | 2 | 6 | 5 | 5 | 6 | 1 | 15 |
| GH4 | α-glucosidase, α-galactosidase, α-glucuronidase and others |  |  |  |  |  |  |  |  |  |  |  |  |  | 1 |  | 1 | 1 |  |  |  |  | 2 | 2 | 2 |  | 1 |
| GH5 | endo-β-1,4-glucanase, cellulose and others |  |  |  |  |  |  |  |  | 1 |  |  |  |  |  |  |  |  |  |  |  | 1 |  |  |  | 2 |  |
| GH13 | α-amylase and related enzymes | 3 | 2 | 1 | 4 | 5 | 4 | 3 | 4 | 1 | 4 | 5 | 2 | 2 | 4 | 4 | 5 | 4 | 2 | 2 | 3 | 6 | 5 | 5 | 6 | 2 | 9 |
| GH16 | chitin β-1,6-glucanosyltransferase and others | 1 |  |  | 1 |  | 1 |  |  |  | 3 | 2 |  | 1 |  |  |  | 1 | 1 | 1 | 1 |  |  |  |  | 1 | 3 |
| GH18 | chitinase;lysozyme, others | 1 |  |  |  | 2 | 2 |  | 1 |  | 1 |  |  | 1 | 1 | 2 | 3 | 1 | 1 | 3 | 2 | 1 | 4 | 3 | 3 | 3 | 3 |
| GH23 | G-type lysozyme, peptidoglycan lyase, peptidoglycan-lytic transglycosylase | 1 | 2 | 1 | 2 | 15 | 3 |  | 2 | 12 | 5 | 12 | 4 | 4 | 9 | 7 | 9 | 3 | 5 | 5 | 7 | 11 | 12 | 13 | 1 | 4 | 3 |
| GH43 | xylanase and others | 5 | 5 | 2 |  |  | 2 |  | 7 |  |  |  |  |  |  |  | 2 |  |  |  |  | 1 | 1 | 1 |  |  | 6 |
| GH53 | endo-β-1,4-galactanase |  |  |  |  |  |  | 5 |  | 3 | 3 | 2 | 1 | 7 | 1 |  | 5 | 5 |  | 24 | 26 | 2 | 23 | 23 | 22 | 21 | 43 |
| GH93 | exo-α-L-1,5-arabinase |  |  |  |  |  |  |  |  | 1 | 1 | 1 | 1 |  | 3 | 1 |  |  |  |  |  |  |  |  |  |  |  |
| GH109 | α-N-acetylgalactosaminidase |  |  |  |  | 2 | 1 |  |  |  |  |  |  |  |  |  |  | 1 |  |  | 1 | 2 | 1 | 1 | 1 | 1 | 1 |
| GH130 | 1-β-D-mannopyranosyl-4-D-glucopyranose: phosphate α-D-mannosyltransferase |  |  |  |  | 1 |  | 1 |  |  | 1 | 1 | 1 |  |  | 1 |  |  |  |  |  |  | 3 | 3 | 3 |  | 2 |
| PL1 | pectate lyase; exo-pectate lyase; pectin lyase | 1 |  |  |  |  |  |  | 1 |  |  |  |  |  |  |  | 1 |  |  |  |  |  |  |  |  |  |  |
| PL9 | pectate lyase; exopolygalacturonate lyase; thiopeptidoglycan lyase |  |  |  |  | 1 |  |  |  |  | 3 | 5 |  |  |  |  |  |  |  |  |  |  |  |  |  |  |  |
| PL22 | oligogalacturonate lyase; oligogalacturonide lyase |  |  |  |  |  |  |  |  |  | 1 | 1 | 1 |  |  |  |  |  |  |  |  |  |  |  |  |  |  |
|  |  | **27** | **28** | **29** | **30** | **31** | **32** | **33** | **34** | **35** | **36** | **37** | **38** | **39** | **40** | **41** | **42** | **43** | **44** | **45** | **46** | **47** | **48** | **49** | **50** | **51** |  |
| GH1 | β-glucosidase, β-galactosidase and others | 18 | 12 | 4 | 5 | 7 | 7 | 6 | 6 | 5 | 1 | 4 | 1 | 1 | 1 | 3 | 4 |  | 2 | 11 | 11 | 8 | 2 | 3 | 2 | 3 |  |
| GH2 | β-galactosidase, β-glucuronidase, β-mannosidase and others | 2 |  | 1 |  | 2 | 1 | 1 | 2 | 2 |  | 1 | 1 | 1 |  |  | 1 |  |  |  |  | 1 |  | 1 |  |  |  |
| GH3 | β-glucosidase, xylan 1,4-β-xylosidase | 7 | 4 | 5 | 1 | 2 | 14 | 7 | 13 | 7 | 4 | 1 | 3 | 4 | 5 | 5 | 1 | 1 |  | 3 | 5 | 2 |  | 2 | 1 |  |  |
| GH4 | α-glucosidase, α-galactosidase, α-glucuronidase and others | 1 |  |  | 1 | 1 |  |  | 1 |  |  | 1 |  |  | 1 |  |  |  |  |  |  | 2 |  |  |  |  |  |
| GH5 | endo-β-1,4-glucanase, cellulose and others |  |  |  |  |  | 1 |  |  |  | 2 | 1 |  | 1 | 1 |  |  |  |  |  |  |  |  |  |  |  |  |
| GH13 | α-amylase and related enzymes | 9 | 3 | 1 | 2 | 3 | 8 | 4 | 1 | 12 | 2 | 5 | 2 | 3 | 3 | 1 | 4 |  | 2 | 4 | 1 | 4 |  | 6 | 4 | 2 |  |
| GH16 | chitin β-1,6-glucanosyltransferase and others | 1 |  | 2 |  |  |  | 4 | 3 | 1 | 3 |  |  | 1 |  | 1 |  |  |  | 2 |  |  |  |  | 7 |  |  |
| GH18 | chitinase;lysozyme and others | 4 | 2 | 1 | 1 |  | 2 | 2 | 3 | 3 | 3 | 1 |  | 1 | 1 | 5 | 1 |  |  | 17 | 19 |  |  |  | 1 | 2 |  |
| GH23 | G-type lysozyme, peptidoglycan lyase, peptidoglycan-lytic transglycosylase | 28 | 2 | 3 | 5 | 7 | 6 | 6 | 2 | 17 | 5 | 5 | 1 | 6 | 2 | 2 | 2 |  |  | 5 | 5 | 1 |  |  | 2 |  |  |
| GH43 | xylanase and others | 2 |  | 2 | 5 |  | 5 |  | 1 |  |  | 1 |  |  |  |  |  |  |  |  |  | 1 |  |  | 1 |  |  |
| GH53 | endo-β-1,4-galactanase | 16 | 5 | 14 | 11 | 6 |  | 18 | 35 | 37 | 14 |  |  | 2 | 2 | 1 | 2 | 2 | 1 | 2 | 2 |  |  | 2 | 18 |  |  |
| GH93 | exo-α-L-1,5-arabinase | 1 |  |  | 1 |  |  | 1 |  |  |  |  |  |  | 1 |  |  |  |  |  |  |  |  |  |  |  |  |
| GH109 | α-N-acetylgalactosaminidase |  | 1 | 1 | 1 | 2 | 2 | 1 | 2 | 1 |  | 1 |  |  | 2 |  | 2 |  |  | 1 | 1 | 1 |  |  |  |  |  |
| GH130 | 1-β-D-mannopyranosyl-4-D-glucopyranose: phosphate α-D-mannosyltransferase | 1 |  |  |  | 1 | 1 |  | 1 | 5 | 1 | 1 |  |  |  |  |  |  |  | 1 |  | 1 |  |  | 1 |  |  |

**Table S2.** Summary of the related metagenomics and metatranscriptomic Illumina sequencing datasets of the two biofilm samples.

| **Data** | **Type** | **Clean reads (paired-end reads)** | **Length of reads (bp)** | **Source** |
| --- | --- | --- | --- | --- |
| biofilm_Al_  metagenome | Illumina HiSeq2000 | 44,146,969 | 100 | Zhang et al., 2015 |
| biofilm_PVC_  metagenome | Illumina HiSeq2000 | 39,229,148 | 100 | Zhang et al., 2015 |
| biofilm_Al_  metatranscriptome | Illumina HiSeq2000 | 3,255,948 | 100 | This study |
| biofilm_PVC_  metatranscriptome | Illumina HiSeq2000 | 3,392,912 | 100 | This study |
